# Green Tea Polyphenol (−)-Epigallocatechin-3-Gallate Restores Nrf2 Activity and Ameliorates Crescentic Glomerulonephritis

**DOI:** 10.1371/journal.pone.0119543

**Published:** 2015-03-18

**Authors:** Ting Ye, Junhui Zhen, Yong Du, Jason K. Zhou, Ai Peng, Nosratola D. Vaziri, Chandra Mohan, Yan Xu, Xin J. Zhou

**Affiliations:** 1 Department of Clinical Nutrition, Tongji Hospital, Tongji Medical College, Huazhong University of Science and Technology, Wuhan, Hubei, China; 2 Department of Pathology, University of Texas Southwestern Medical Center, Dallas, Texas, United States of America; 3 Department of Pathology, Shandong University School of Medicine, Jinan, Shandong, China; 4 Department of Biomedical Engineering, University of Houston, Houston, Texas, United States of America; 5 Division of Rheumatology, University of Texas Southwestern Medical Center, Dallas, Texas, United States of America; 6 Center for Nephrology and Clinical Metabolomics, Department of Nephrology & Rheumatology, Shanghai Tenth People's Hospital, Tongji University School of Medicine, Shanghai, China; 7 Division of Nephrology and Hypertension, University of California Irvine, Irvine, California, United States of America; 8 Department of Nephrology, Qingdao University Affiliated Hospital, Qingdao, Shandong, China; 9 Renal Path Diagnostics, Pathologist BioMedical laboratories, Lewisville, Texas, United States of America; 10 Department of Pathology, Baylor University Medical Center, Dallas, Texas, United States of America; Winship Cancer Institute of Emory University, UNITED STATES

## Abstract

Crescentic glomerulonephritis (GN) is the most severe form of GN and is associated with significant morbidity and mortality despite aggressive immunotherapy with steroids, cytotoxic drugs, and plasmapheresis. We examined the therapeutic efficacy of the green tea polyphenol (−)-epigallocatechin-3-gallate (EGCG, 50 mg/kg BW/day x3weeks), a potent anti-inflammatory and anti-oxidant agent, on experimental crescentic GN induced in 129/svJ mice by administration of rabbit anti-mouse glomerular basement membrane sera. Routine histology and key molecules involved in inflammatory and redox signaling were studied. EGCG treatment significantly reduced mortality, decreased proteinuria and serum creatinine, and markedly improved renal histology when compared with vehicle-treated mice. The improvements in renal function and histology were accompanied by the restoration of Nrf2 signaling (which was impaired in vehicle-treated mice) as shown by increased nuclear translocation of Nrf2 and cytoplasmic glutamate cysteine ligase catalytic subunit, glutamate cysteine ligase modifier subunit, and glutathione peroxidase. EGCG-treated mice also showed reduction in p-Akt, p-JNK, p-ERK1/2 and p-P38 as well as restoration of PPARγ and SIRT1 levels. Lower dose of EGCG (25 mg/kg BW/day x2 weeks) treatment also significantly decreased proteinuria and serum creatinine, and markedly improved renal histology when compared with vehicle-treated mice. Thus, our data illustrate the efficacy of EGCG in reversing the progression of crescentic GN in mice by targeting multiple signaling and inflammatory pathways as well as countering oxidative stress.

## Introduction

Crescentic glomerulonephritis (GN) includes a variety of conditions characterized by glomerular fibrinoid necrosis and accumulation of cells in Bowman’s space. It can be classified into three categories: pauci-immune, immune complex-mediated, and anti-glomerular basement membrane (GBM) antibody-induced crescentic GN (anti-GBM-GN) [1,2]. Anti-GBM-GN is pathologically and clinically the most severe form of GN with end-stage renal disease developing in 40–70% of the affected patients [1,2]. It is caused by an inflammatory reaction in the glomerular capillaries initiated by circulating antibodies directed to the GBM components, non-collagenous-1 (NC1) domain of the α3 or α5 chain of type IV collagen [1,3,4]. The contemporary treatment of anti-GBM-GN aims to modulate the injury-causing immunologic process with high-dose corticosteroids, cytotoxic drugs, and plasmapheresis. However, the nonspecific nature of these therapeutic regimes and frequently disabling side effects beg for an urgent development of new and more targeted therapeutic strategies [5].

Oxidative stress and inflammation play major roles in the pathogenesis and progression of acute and chronic kidney diseases. Overproduction of reactive oxygen species (ROS), reactive nitrogen species, and reactive chlorine species by inflammatory cells can cause tissue damage, intensify inflammation, promote apoptosis, and accelerate progression of many diseases including anti-GBM-GN [6]. Nuclear factor erythroid 2-related factor 2 (Nrf2)/Kelch-like ECH-associated protein 1 (KEAP1) complex is used by the cells to detect and respond to chemical and oxidative stresses. Through oxidation of the sulfhydryl groups in the cysteine residues of KEAP1, oxidative and electrophilic stress limit its ability to bind Nrf2 and thereby enhance its translocation to the nucleus, where it binds to the antioxidant response element (ARE) in the promoter regions of numerous genes encoding antioxidant and cytoprotective enzymes and proteins [7]. This leads to increased production of phase 2 detoxifying enzymes such as glutathione-S-transferases and NAD(P)H:quinone oxidoreductase 1 (NQO1) and antioxidant enzymes such as heme oxygenase 1 (HO1) and glutathione synthetic enzymes [8–10]. Impaired Nrf2 activation was shown to contribute to oxidative stress and inflammation and the progression of tissue damage in rat models of chronic renal failure [11]. Similarly, progressive focal glomerulosclerosis in a spontaneous rat model is associated with oxidative stress, inflammation, and impaired Nrf2 activation [12]. In addition, Nrf2 gene ablation has been shown to cause lupus-like autoimmune nephritis [13].

The green tea catechins, particularly (-)-epigallocatechin-3-gallate (EGCG), are potent anti-inflammatory and anti-oxidant agents shown to inhibit leukocyte chemotaxis, quench free radicals, chelate transition metals, and interrupt lipid peroxidation chain reaction [14]. It has been shown that EGCG upregulates Nrf2 signaling and ameliorates cisplatin-induced acute kidney injury in rats and lupus nephritis in mice [15, 16]. We have previously shown that prophylactic pretreatment with EGCG favorably affects the course of crescentic GN in a murine model of anti-GBM-GN by targeting redox and inflammatory pathways [17]. However, its effectiveness in treating full-blown crescentic GN and the potential mechanisms have not been fully elucidated. In addition, the effect of anti-GBM GN on Nrf2 pathway is unknown. In the present study, we tested the hypothesis that EGCG can be an effective therapeutic agent for crescentic GN by administration of EGCG to male 129/svJ mice with anti-GBM antibody-induced GN.

## Materials and Methods

### Animal model and experimental design

All studies were reviewed and approved by the institutional review committee at UT Southwestern. Anti-GBM serum was generated by Lampire Laboratories (Pipersville, PA, USA) [18, 19]. Pre-immune rabbit serum was used as negative control. Anti-GBM-GN was induced in male 129/svJ mice (Jackson Laboratories, Bar Harbor, ME, USA) [18, 19]. Briefly, 40 eight-week old mice were pre-sensitized on day-5 (5 days before inducing anti-GBM-GN) with rabbit IgG (140 μg per mouse) in complete Freund’s adjuvant (Sigma-Aldrich, St. Louis, MO, USA). On day 0, the mice received anti-GBM serum; 140 μg of total IgG in a 100 μl volume was administered intravenously. All mice were maintained in a specific pathogen-free colony.

To explore the therapeutic effect of EGCG (Sigma-Aldrich), the anti-GBM antibody-injected mice were allowed to develop full-blown nephritis for 7 days before intervention [17–19]. On day 7 following anti-GBM antibody administration, the mice were randomized into either EGCG- or vehicle-treated group. The former group (anti-GBM/EGCG, n = 18) received 50 mg/kg BW/day of EGCG orally (gavage, dissolved in normal saline in a total volume of 100 ul) [17, 20] for 3 weeks (day 7 to day 28) until sacrifice on day 29. Normal saline was given to the vehicle group (anti-GBM/Vehicle, n = 22) with the same protocol. A group of mice (Control, n = 10) without anti-GBM antibody or EGCG administration was included as normal control. On day-5 (baseline), day 7, and day 28, sera and 24-hour urine samples were collected from all mice using metabolic cages with free access to drinking water. Following pre-sensitization, mice were checked twice daily and weighed thrice per week. At the end of experiments (day 29) or when predetermined humane endpoints were reached, animals were euthanized via CO2 inhalation followed by cervical dislocation. Mice were humanely euthanized if they met 2 or more of the following criteria: hunched posture, blood present in the urine and/or faces, weight loss = />20% of baseline level or = />10% in the previous 24h, piloerection and lack of response to external stimuli. Analgesics were not administered as they could interfere with the inflammatory process. Blood was collected by cardiac puncture and the kidneys were processed for further analyses as described below.

### Measurement of urine protein and serum creatinine

All urine samples were centrifuged at 14,000 rpm for 5 min. Clear supernatant was used for urine protein assay (Coomassie Plus protein assay kit, Cat. # 23236, Pierce, Rockford, IL, USA). The serum creatinine levels were measured as previously described [21].

### Measurement of systemic immune response to injected anti-GBM antibodies

Mouse antibodies to rabbit immunoglobulin were assayed by ELISA as described previously [19]. Briefly, purified rabbit Ig (Sigma-Aldrich) was coated onto Immulon I plates (Dynatech, Chantilly, CA, USA) and then blocked. Serially diluted mouse sera were added to the plates and the bound mouse anti-rabbit Ig was detected using alkaline-phosphatase-conjugated goat anti-mouse IgG (Roche, Indianapolis, IN, USA) that did not cross-react with rabbit Ig and P-nitrophenyl phosphate substrate (Sigma-Aldrich). Color development was measured spectrophotometrically at 405 nm. All assays were run in duplicate, and samples were reanalyzed when standard errors >10% were found.

### Renal histopathology and immunohistochemistry

Three-micrometer sections of formalin-fixed and paraffin-embedded (FFPE) kidney tissues were cut and stained with hematoxylin and eosin (H&E) and periodic acid-Schiff (PAS) reaction. These sections were examined in a blinded fashion. Glomerulonephritis was graded on 0–4 scale and tubulointerstitial injury was graded on a 0–5 scale, as detailed previously [17, 19, 22]. The numbers of intra-renal leukocytes within the glomeruli and interstitium were counted by staining FFPE tissue sections with antibodies to lymphocytes (CD3, Cat. # MCA1477, 1:500, AbD Serotec, Raleigh, North Carolina, USA) and macrophages (Iba1, Cat. # 019–19741, 1:500, Wako Chemicals, Richmond, VA, USA). Standard avidin-biotin complex (ABC) method was used for immunohistochemical staining [17, 22].

### Measurement of markers of oxidative stress and antioxidant system

Lipoperoxides in serum, urine, and renal tissue were determined by measurement of malondialdehyde-thiobarbituric acid with a TBARS assay kit (Cat. no. 10009055; Cayman Chemical Company, Ann Arbor, MI, USA), according to the manufacturer’s protocol. Total glutathione (GSH) content of the renal tissue was determined using a Cayman GSH assay kit (Cayman Chemical Co). The assay uses a carefully optimized enzymatic recycling method using GSH reductase whereby the sulfhydryl group of GSH reacts with 5,5′-dithio-bis-2-nitrobenzoic acid and Ellman's reagent producing a yellow colored 5-thio-2-nitrobenzoic acid (TNB). The mixed disulfide, GSTNB (between GSH and TNB), which is concomitantly produced, is reduced by GSH reductase to recycle GSH and produce more TNB. The rate of TNB production is directly proportional to this recycling reaction, which is in turn directly proportional to the concentration of GSH in the sample. Thus, measurement of TNB at 405 or 412 nm provides an accurate estimate of GSH present in the sample. It should be noted that oxidized GSH is converted to GSH by GSH reductase in this system, which consequently measures total GSH.

### Western Blot Analyses

The renal tissue was homogenized and total protein was prepared using RIPA Lysis and Extraction Buffer (Thermo Scientific, Waltham, MA, USA). The nuclear extract was prepared with NE-PER Nuclear and Cytoplasmic Extraction Reagent kit (Thermo Scientific). Protein content was quantified using BCA Protein Assay Reagent (Thermo Scientific). For measurement of protein expression, western blotting was carried out as previously described [17, 23]. Briefly, 40 μg protein was size-fractionated on 4–12% Tris-glycine gel (Biorad, Hercules, CA, USA) at 100 V for 2 h. After electrophoresis, the proteins were transferred onto nitrocellulose membrane (Bio-rad) at 350 mA for 2.5 h. The membrane was prehybridized in 10 ml blocking buffer (1xTBS, 0.1% Tween-20, and 5% nonfat milk powder) for 1 h and then hybridized overnight at 4°C with the following primary antibodies (diluted with the same buffer): nuclear factor-erythroid-2-related factor 2 (Nrf2, ab31163, 1:5000, Abcam, Cambridge, MA, USA), glutamate-cysteine ligase catalytic subunit (GCLc, ab41463, 1:5000, Abcam), glutamate-cysteine ligase modifier subunit (GCLm, ab81445, 1:5000, Abcam), glutathione peroxidase (GPx1, ab22604, 1:5000, Abcam), NAD(P)H:quinone oxidoreductase 1 (NQO1, ab34173, 1:5000, Abcam), heme oxygenase-1 (HO-1, ab13243, 1:5000, Abcam), phospho-Akt (Ser473) (# 4058, 1:1000, Cell Signaling, Danvers, MA, USA), phospho-p44/42 MAPK (ERK1/2) (Thr202/Tyr204) (# 9101, 1: 1000, Cell Signaling), phospho-SAPK/JNK (Thr183/Tyr185) (#9251, 1:1000, Cell Signaling), phospho-p38 MAPK (Thr180/Tyr182) (#9211, 1:1000, Cell Signaling), silent information regulator 2 (Sir2) protein 1 (SIRT1, ab12193, 1:2000, Abcam), peroxisome proliferator-activated receptor-gamma (PPARγ, ab19481, 1:1000, Abcam), β-actin (#4967, 1:5000, Cell Signaling) and Lamin A (sc-20680, 1:5000, Santa Cruz Biotechnology, Dallas, TX, USA). Then, the membrane was washed for 30 min in a shaking bath (the wash buffer, TBST, was changed every 10 min for 3 times) and then incubated for 1 h with blocking buffer plus anti-mouse IgG or anti-rabbit IgG tagged with horseradish peroxidase at a final titer of 1:5000. The washes were repeated before the membrane was developed with a light emitting nonradioactive method using ECL reagent (SuperSignal West Dura Kit, Thermo Scientific). The membrane was then subjected to autoluminography. Sometimes, the membrane was stripped with Restore Plus Western Blot Stripping Buffer (Thermo Scientific) and used to measure another target protein. The respective band intensities were measured using Scion Image (WinB403; http://rsb.info.nih.gov/nih-image/).

### Effect of low dose EGCG on renal function and pathology

To examine the effects of low dose EGCG on renal function and pathology, subgroups of anti-GBM antibody-injected mice were allowed to develop full-blown nephritis for 7 days as described previously. On day 7 following anti-GBM antibody administration, the mice were randomized into either EGCG- or vehicle-treated group. The former group (anti-GBM/EGCG, n = 10) received 25 mg/kg BW/day of EGCG orally (gavage) for 2 weeks (day 7 to day 21) until sacrifice on day 22. Normal saline was given to the vehicle group (anti-GBM/Vehicle, n = 10) with the same protocol. A group of mice (Control, n = 7) without anti-GBM antibody or EGCG administration was included as normal control. Sera and 24-hour urine samples (at baseline and on day 21) were collected from all mice using metabolic cages with free access to drinking water. All animals were sacrificed on day 22. Renal function and histopathology were evaluated as described previously.

### Statistical Analyses

Data are presented as mean ± SEM. Analysis of variance (ANOVA), Fisher exact test, and Student’s t-test were used in statistical evaluation of the data as appropriate. P-values less than or equal to 0.05 were considered significant.

## Results

### Disease phenotypes

At the end of week 1 following anti-GBM serum administration, the mice exhibited renal insufficiency and proteinuria. At the end of the 4-week observation period, the vehicle-treated group suffered a mortality rate of 27% compared with 11% in the EGCG-treated group. The vehicle-treated mice with anti-GBM-GN developed renal insufficiency and proteinuria. The EGCG-treated mice showed significantly less proteinuria and lower serum creatinine than the vehicle-treated mice (Table 1). These findings demonstrated that EGCG administration improved renal function and reduced mortality in mice with established anti-GBM GN.

**Table 1:** General and Biomedical Data.

	Serum creatinine (mg/dl)			Proteinuria (mg/24h)			Mortality rate
	Baseline	Day 7	Day 28	Baseline	Day 7	Day 28	
**Control (n = 10)**	**0.071 ± 0.004**	**0.075 ± 0.005**	**0.084 ± 0.007**	**0.65 ± 0.09**	**0.70 ± 0.13**	**0.67 ± 0.12**	**0% (0/10)**
**Anti-GBM/Vehicle (n = 22)**	**0.069 ± 0.003**	**0.162 ± 0.014** *	**0.321 ± 0.03** *	**0.71 ± 0.11**	**13.02 ±1.17** *	**15.62 ± 1.5** *	**27% (6/22)** *
**Anti-GBM/EGCG (n = 18)**	**0.072 ± 0.004**	**0.144 ± 0.013** *	**0.184 ± 0.038** * ^#^	**0.66 ± 0.13**	**11.32 ± 1.55** *	**9.58 ± 1.63** * ^#^	**11% (2/18)** * ^#^

Values are mean ± SEM

Anti-GBM/Vehicle: Vehicle started 7 days after induction of anti-glomerular basement

membrane glomerulonephritis

Anti-GBM/EGCG: (-)-epigallocatecin-3-gallatetreatement started 7 days after the induction of

anti-glomerular basement membrane glomerulonephritis

*P<0.01 vs. normal control

^#^P<0.05 vs. Anti-GBM/vehicle

### Histopathologic data

**Fig 1:**
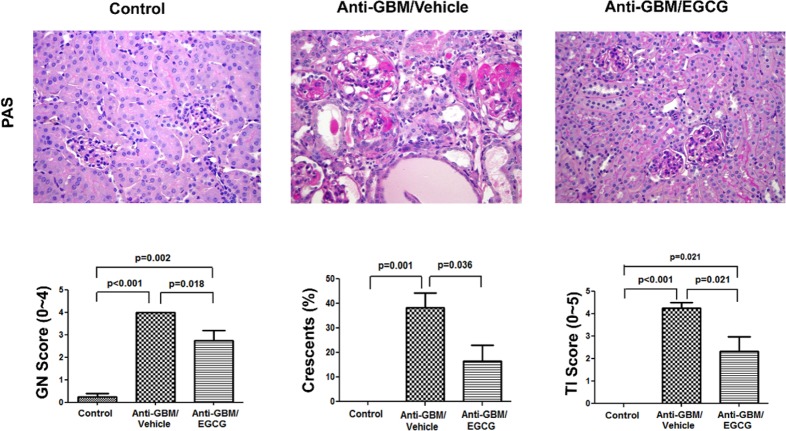
Renal histology of normal control mice, vehicle-treated mice with anti-GBM-GN, and EGCG-treated mice with anti-GBM-GN. Kidneys from mice 4 weeks after induction of anti-GBM-GN with or without EGCG treatment and kidneys from normal control mice were evaluated by light microscopy. In the PAS stained sections, the kidneys in the vehicle-treated mice revealed severe glomerular hypercellularity with large crescents. There were also significant tubular atrophy and interstitial fibrosis with prominent interstitial inflammatory infiltrates (original magnification x 400). In contrast, the EGCG-treated animals exhibited mild to moderate glomerular injury with minimal crescent formation and focal mild tubulointerstitial injury. No significant histopathologic changes were observed in the normal control mice. The bar graphs represent semi-quantification of glomerular in jury (GN score), crescent formation and tubulointerstitial injury (TI score). Values are mean ± SEM.; n = 8–10 in each group.

**Fig 2:**
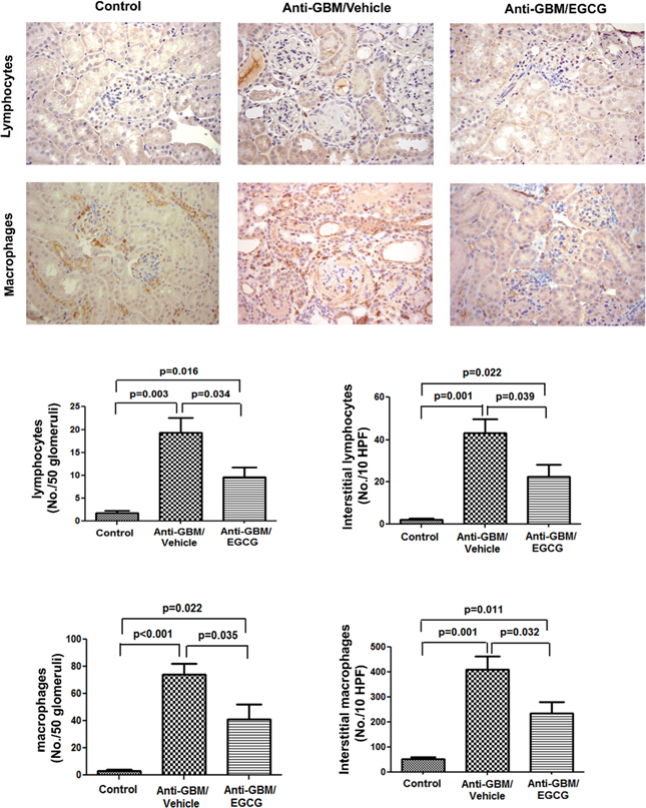
Immunohistochemistry for inflammatory cells in kidneys of normal control mice, vehicle-treated mice with anti-GBM-GN, and EGCG-treated mice with anti-GBM-GN. Kidneys from mice 4 weeks after induction of anti-GBM-GN with or without EGCG treatment and kidneys from normal control mice were evaluated for inflammatory cell infiltration. Immunohistochemical analyses showed markedly increased interstitial infiltration of lymphocytes and macrophages in the vehicle-treated group (original magnification x 400). In contrast, the EGCG-treated animals exhibited significantly less interstitial infiltration of lymphocytes and macrophages. No significant inflammatory cell infiltrates were observed in the normal control mice. The number of lymphocyte and macrophages in glomeruli and interstitial compartment are depicted in the bar graphs. Values are mean ± SEM; n = 8–10 in each group.

The vehicle-treated mice showed moderate to severe renal injury characterized by crescent formation with significant intracapillary hypercellularity, obliterated capillary lumens, and thickened capillary walls. Tubular atrophy and dilation with hyaline casts and interstitial fibrosis were also noted. In comparison, the EGCG-treated mice exhibited milder renal injury with only occasional crescent formation and focal tubulointerstitial injury (Figs. 1 and 2). No lesions were seen in the normal control group. In addition, the vehicle-treated mice showed heavy glomerular and interstitial infiltrations by macrophages and lymphocytes, which was significantly attenuated by EGCG administration (Figs. 1 and 2). Thus, the severe renal lesions and heavy inflammation associated with anti-GBM disease were greatly reduced in by EGCG administration.

### Systemic immune response to administered anti-GBM antibodies

**Fig 3:**
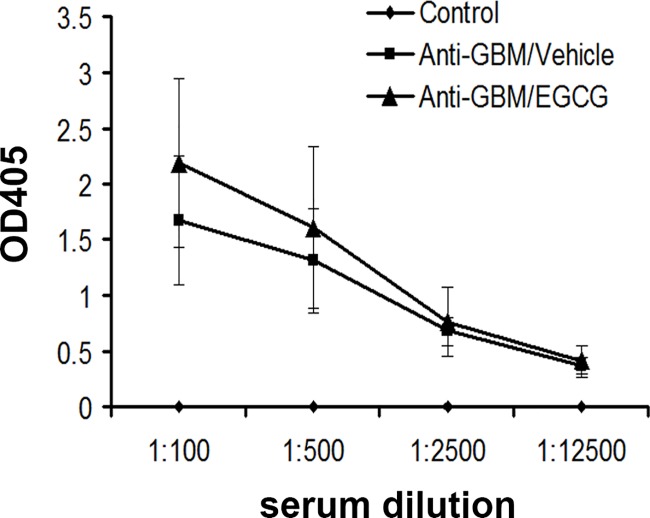
Anti-rabbit Immunoglobulin (Ig) response in EGCG- or vehicle-treated mice with anti-GBM glomerulonephritis. The serum levels of IgG mouse anti-rabbit antibodies (measured on day 28) assayed in serial dilutions are shown. Values are expressed as mean ± SEM; n = 8–10 in each group.

We asked if the reduced renal disease in EGCG treated mice may be due to a decreased xenogenic immune response to the injected rabbit Ig. To answer this question, we compared the levels of IgG mouse anti-rabbit antibodies on day 28 using ELISA. As depicted in Fig. 3, the IgG mouse anti-rabbit antibody titers were comparable between the two groups of mice with anti-GBM-GN, indicating that the amelioration of renal disease in EGCG-treated mice was not caused by a decreased xenogenic immune response to the injected immunoglobulin.

### Markers of oxidative stress

**Fig 4:**
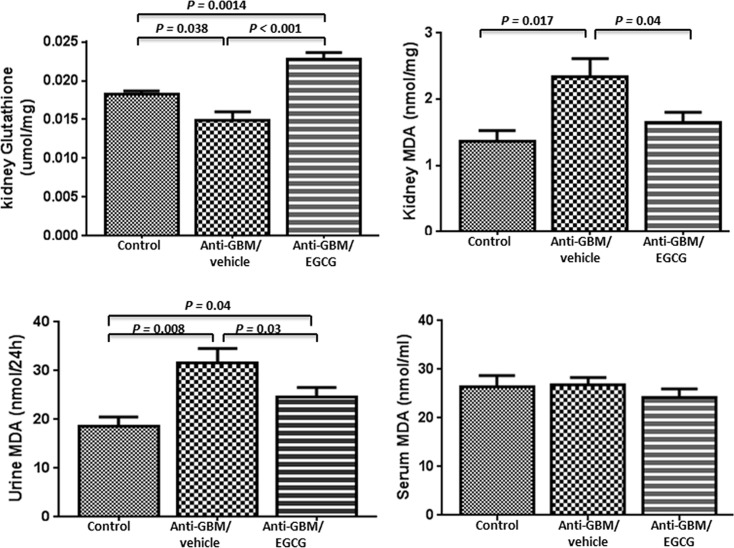
Malondialdehyde (MDA) and glutathione (GSH) in mice with anti-glomerular basement membrane antibody-induced glomerulonephritis (anti-GBM-GN). Urine and renal tissue MDA levels were markedly elevated in mice with anti-GBM glomerulonephritis compared with the normal controls. Treatment with EGCG normalized the urine and renal tissue MDA levels. Serum MDA levels were not significantly altered among the three groups. Renal tissue GSH levels were modestly reduced in mice with anti-GBM glomerulonephritis compared with the normal controls. Treatment with EGCG increased the renal tissue GSH to supranormal levels. Values are expressed as mean ± SEM; n = 7–9 in each group.

Renal tissue and urine malondialdehyde (MDA) levels were modestly higher in the vehicle-treated mice than the normal control group. EGCG treatment normalized the MDA levels in both renal tissue and urine. Serum MDA levels were not significantly different among the three groups (Fig. 4). Renal tissue glutathione levels were modestly lower in the vehicle-treated mice than the normal control group. EGCG treatment markedly elevated the tissue glutathione to a supranormal level (Fig. 4). These findings point to heightened ROS-induced lipid peroxidation and glutathione oxidation in the animals with anti-GBM GN. EGCG treatment reduced oxidative stress associated with anti-GBM-GN.

### Nrf2 signaling data

**Fig 5:**
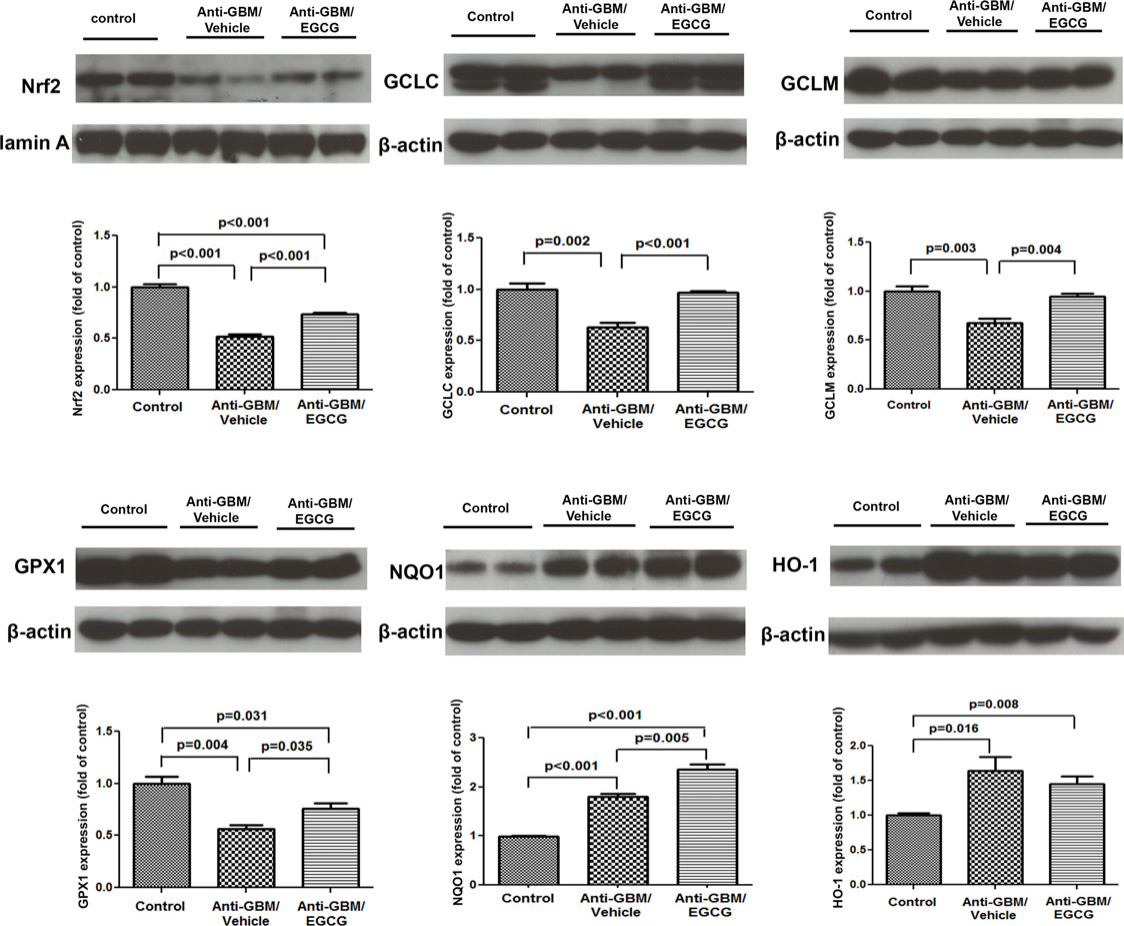
Western Blot analyses of Nrf2 signaling in kidneys of normal control mice, vehicle-treated mice with anti-GBM-GN, and EGCG-treated mice with anti-GBM-GN. Western blot showed significant reduction in the levels of Nrf2, GCLc, GCLm, and GPx1 in vehicle-treated mice with anti-GMB-GN, compared to the levels in normal control mice. EGCG treatment of mice with anti-GBM-GN led to significant increase in the levels of Nrf2, GCLc, GCLm, and GPx1. In contrast, vehicle-treated mice with anti-GMB-GN showed significantly increased levels of NQO1 and HO1, compared to the levels in normal control mice. EGCG treatment further increased the NQO1 levels but did not significantly affect the HO1 levels, compared to the levels in vehicle-treated mice. Values are expressed as mean ± SEM; n = 8–10 in each group.

Compared with the normal control group, the vehicle-treated anti-GBM-GN mice showed a significant reduction in nuclear Nrf2 abundance, pointing to impaired Nrf2 activation. This was associated with significant reduction of GCLc, GCLm, and GPx-1 that are important products of Nrf2-regulated genes (Fig. 5). Surprisingly, despite impaired Nrf2 signaling, the expression of NQO1 and HO1 that are also regulated by Nrf2 was elevated in the vehicle-treated mice. Treatment with EGCG led to partial restoration of nuclear Nrf2 protein content and cytoplasmic GCLc, GCLm, and GPx1 protein abundance, further increase in NQO1 level, and a slight reduction in HO1abundance in the renal cortex of the anti-GBM-GN mice. Taken together, these data demonstrated impaired Nrf2 activation in mice with anti-GBM GN and EGCG treatment restored Nrf2 signaling.

### PI3k/Akt and MAPK data

**Fig 6:**
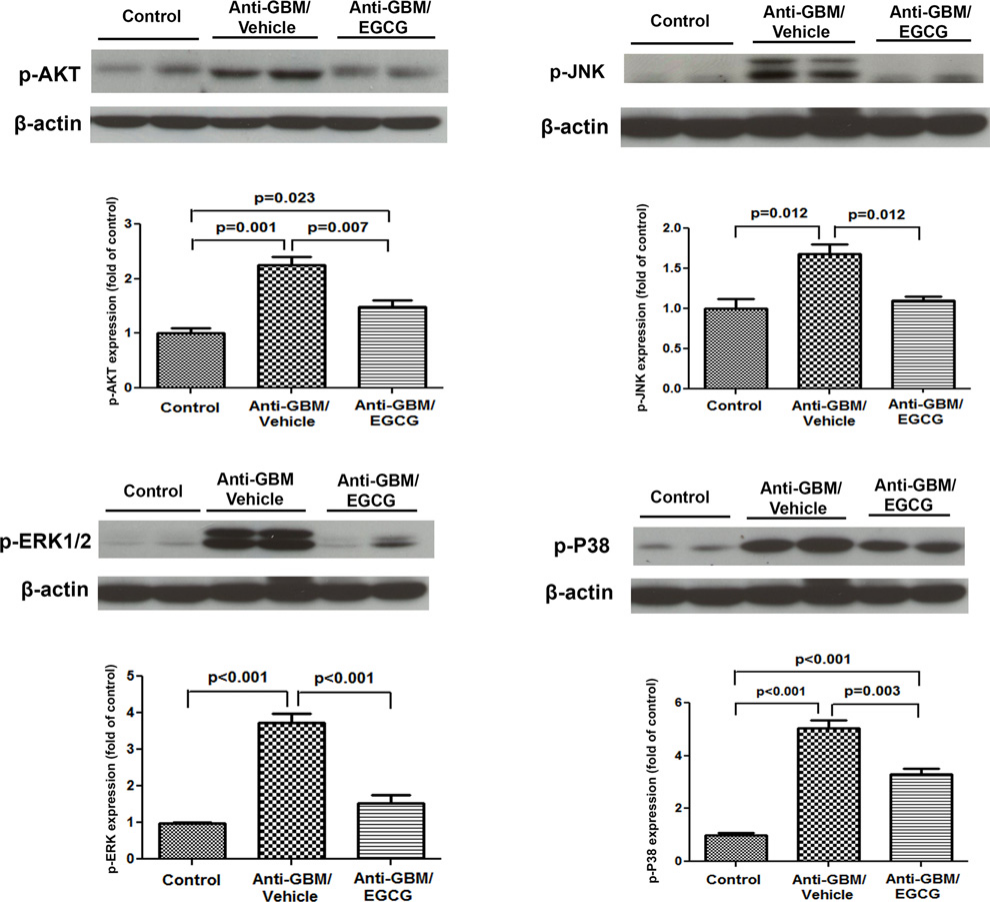
Western Blot analyses of PI3k/Akt and MAPK signaling in kidneys of normal control mice, vehicle-treated mice with anti-GBM-GN, and EGCG-treated mice with anti-GBM-GN. Western blot showed significant increase in the levels of p-Akt, p-JNK, p-ERK, and p-P38 in vehicle-treated mice with anti-GMB-GN, compared to the levels in normal control mice. EGCG treatment of mice with anti-GBM-GN led to significant reduction in the levels of p-Akt, p-JNK, p-ERK, and p-P38. Values are expressed as mean ± SEM; n = 8–10 in each group.

The vehicle-treated anti-GBM-GN mice exhibited activation of the PI3k/Akt and MAPK signaling pathways in the kidneys as evidenced by significant increase in the p-Akt, p-JNK, p-ERK1/2, and p-P38 levels (Fig. 6). These proteins were significantly reduced by EGCG administration. The observed dampening of these signaling pathways following EGCG administration was likely reflecting the reduced inflammation and oxidative stress.

### PPARγ and SIRT1 data

**Fig 7:**
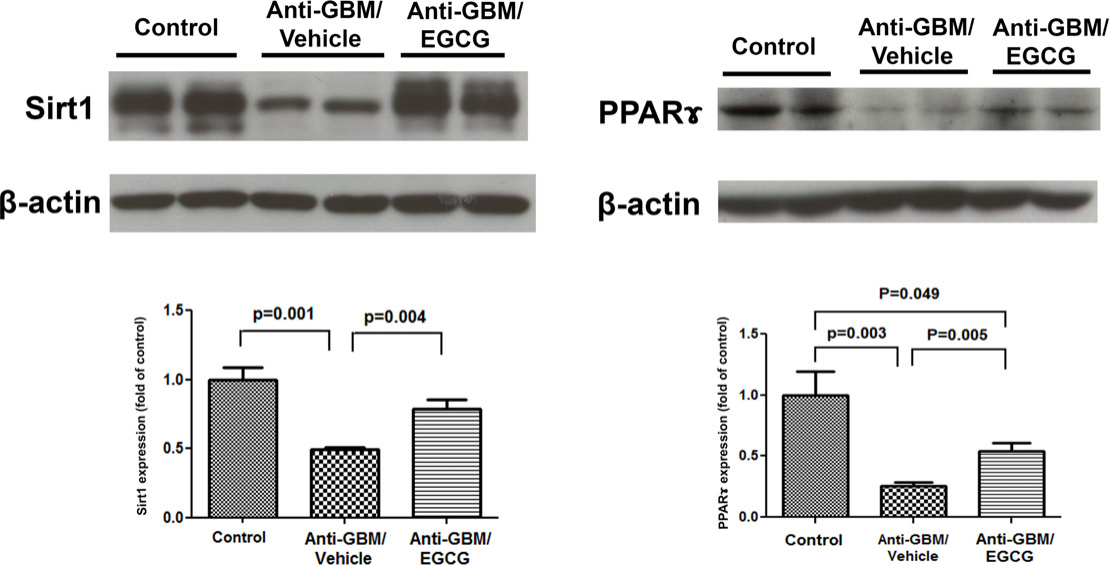
Western Blot analyses of SIRT1 and PPARγ in kidneys of normal control mice, vehicle-treated mice with anti-GBM-GN, and EGCG-treated mice with anti-GBM-GN. Western blot showed significant reduction in the levels of SIRT1 and PPARγ in vehicle-treated mice with anti-GMB-GN, compared to the levels in normal control mice. EGCG treatment of mice with anti-GBM-GN led to significant increase in the levels of SIRT1 and PPARγ. Values are expressed as mean ± SEM; n = 8–10 in each group.

Compared with the normal control group, the vehicle treated anti-GBM-GN mice showed significant reduction of PPARγ and SIRT1 in the kidney tissue. EGCG administration reversed these changes (Fig. 7). Thus, by activating PPARr (an anti-inflammatory transcription factor), EGCG reduces inflammation and by restoring SIRT1 level, EGCG facilitates tissue repair and regeneration.

### Effect of low dose EGCG on renal function and histology

**Fig 8:**
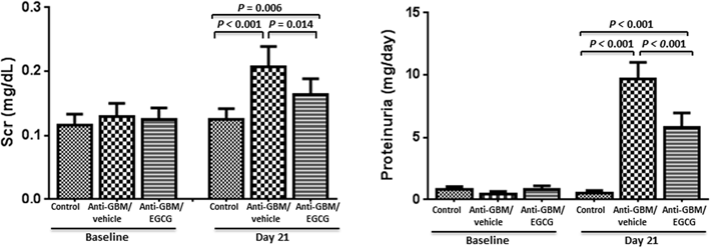
Low dose of EGCG (25 mg/kg BW/day) on renal function. At the end of the 3-week observation period, the vehicle-treated mice with anti-GBM-GN developed renal insufficiency and proteinuria in contrast to the normal control group. The EGCG-treated mice showed significantly less proteinuria and lower serum creatinine than the vehicle-treated mice. Values are expressed as mean ± SEM; n = 7–9 in each group.

At the end of the 3-week observation period (2 weeks post EGCG therapy), two mice were dead in the vehicle-treated group compared with 1 fatality in the EGCG-treated group. The vehicle-treated mice with anti-GBM-GN developed marked renal insufficiency and proteinuria. The EGCG-treated mice showed significantly less proteinuria and lower serum creatinine than the vehicle-treated mice (Fig. 8). In addition, low dose EGCG treatment ameliorated renal injury similar to that observed in high dose EGCG treated group (data not shown). These data demonstrated that low dose EGCG (25 mg/kg B.W) is equally effective in ameliorating renal injury in mice with anti-GBM-GN.

## Discussion

As expected, the untreated mice with anti-GBM-GN exhibited severe renal injury and dysfunction. Treatment with EGCG in mice with established anti-GBM-GN resulted in a dramatic and unequivocal improvement in the biochemical and histological abnormalities as evidenced by significant reductions in proteinuria and serum creatinine, and improvement in histological findings including decreased glomerular crescents, less tubulointerstitial injury, and reduced glomerular and interstitial inflammatory cells. The observed amelioration of renal disease in EGCG-treated mice was not due to a reduction in immune response to the administered rabbit immunoglobulin because the levels of IgG mouse anti-rabbit antibodies were comparable between the vehicle- and EGCG-treated mice with anti-GBM-GN. In agreement with this finding, a recent study showed that EGCG did not change the titers of anti-dsDNA or the glomerular IgG deposition in experimental lupus nephritis even though the renal protective effect is striking [16].

We have previously shown that glomerular disease in the mouse model of anti-GBM-GN is accompanied by oxidative damage as shown by increased levels of malondialdehyde and H_2_O_2_, upregulation of myeloperoxidase and NADPH oxidase, downregulation of catalase and glutathione peroxidase, increased levels of nitrotyrosine, activation of NFkB, upregulation of inducible nitric oxide synthase and osteopontin expression, and decreased PPARγ expression [17]. We further demonstrated that pretreatment with EGCG can attenuate kidney disease and prevent oxidative and nitrosative injury in mice with anti-GBM-GN [17]. The results of the present study extend our earlier observations by demonstrating that EGCG therapy can significantly increase renal glutathione levels and ameliorate established anti-GBM-GN.

The current study, for the first time, demonstrates that the glomerular disease in a mouse model of anti-GBM-GN is associated with impaired Nrf2 activation (reduced nuclear content) along with low levels of GCLc, GCLm, and GPx1. This was accompanied by increased HO1 and NQO1 abundance in the renal tissue, reflecting Nrf2-independent regulation of HO1 and NOQ1 and involvement of other transcriptional regulators in response to inflammatory and oxidative injury as shown by other investigators [24–26]. Treatment with EGCG resulted in marked improvement in renal function and histological lesions as well as reduced mortality in mice with established anti-GBM-GN. This was associated with restoration of Nrf2 activity and of GCLc, GCLm, and GPx1 expressions. Similar results were reported by Sahin et al [15], who found upregulation of the Nrf2 signaling with EGCG administration in rats with cisplatin-induced acute kidney injury. The underlying mechanisms of the reduced Nrf2 activation in the face of severe oxidative stress and inflammation in anti-GBM GN may be multifactorial. The inhibitory effect of NF-kB on Nrf2-ARE pathway plays a major role. We have previously shown that anti-GBM-GN is associated with marked activation of p65/NF-kB molecule [17]. p65/NF-kB interacts with keap1 in the cytoplasm preventing release and translocation of Nrf2 to the nucleus and in the nucleus it prevents binding of Nrf2 to the ARE of its target genes. Together these events enhance Nrf2 ubiquitination and reduce its ability to promote expression of its target gene [27]. Similar phenomenon occurs in chronic kidney disease induced by 5/6 nephrectomy [11] and chronic neurodegenerative diseases [28].

Silent information regulator 2 (Sir2) proteins (Sirtuins) belong to an evolutionary conserved family of NAD^+^-dependent enzymes with deacetylase and/or mono-ADP-ribosyltransferase activity. They regulate DNA repair and recombination, chromosomal stability, gene transcription, and mediate the health-promoting effects of caloric restriction including the retardation of aging. Activation of sirtuins may increase resistance to metabolic, oxidative, and hypoxic stress in different tissues. Of the seven Sir2 homologs in mammals, the most extensively studied is SIRT1, which is cytoprotective in the kidney and participates in the regulation of blood pressure and sodium balance [29]. The anti-GBM-GN mice employed in the present study showed marked reduction of SIRT1 abundance in the renal tissue. Given its important role in the tissue repair and regeneration, the observed downregulation of SIRT1 in the renal tissue of the untreated anti-GBM-GN mice is likely contributed to progression of kidney disease in this model. Administration of EGCG partially restored renal tissue SIRT1 expression in the treated animals, a phenomenon that may have contributed to the salutary effects of this agent.

PPARγ belongs to a family of nuclear hormone receptors with protective role in kidney diseases [30]. PPARγ agonists attenuate nephropathy in experimental models of anti-GBM-GN [31], lupus erythematosus, type 2 diabetes, and non-diabetic glomerulosclerosis, and decrease proteinuria in patients with type 2 diabetes and non-diabetic renal disease [32–35]. We previously showed that PPARγ expression is downregulated in mice with anti-GBM-GN and that PPARγ downregulation is prevented by pretreatment with EGCG. Furthermore, concomitant administration of EGCG with a selective PPARγ antagonist GW9662 prevented the EGCG-induced upregulation of PPARγ and reversed the renoprotective effect of EGCG, pointing to the essential role of the PPARγ pathway in mediating the anti-inflammatory capacity of EGCG [17]. The results of the present study extend our earlier observations by demonstrating the efficacy of EGCG in reversing the downregulation of PPARγ in animals with established nephropathy. Pertinently, it has recently been demonstrated that Nrf2 binds to ARE in the PPARγ promoter, thereby directly activating the transcription of PPARγ [36], suggesting that EGCG-induced PPARγ upregulation in our mice may be mediated by Nrf2. However, further mechanistic studies would be needed to confirm this hypothesis.

Multiple signaling pathways (such as AKT and MAPK/ERK) become activated in various renal diseases and following a wide variety of triggers including oxidative stress and inflammation [37, 38]. EGCG has been shown to inhibit the MAPK pathways and pre-treatment with EGCG has been shown to prevent UVB-induced H_2_O_2_ production and inhibit UVB-induced phosphorylation of ERK1/2, JNK, and P38 in cultured human epidermal keratinocytes [39]. Therefore, restoration of renal PPARγ expression and the salutary effect on kidney function and structure by EGCG may be, in part, related to its ability to attenuate activation of ERK1/2, JNK, and P38. This supposition is consistent with the observed reduction of p-ERK1/2, p-JNK, and p-P38 in EGCG treated mice. Additionally, inhibition of MAPKs by EGCG could potentially retard the PI3K/Akt pathway activation [40], which is consistent with the observed reduction of p-Akt in the EGCG-treated mice kidneys. This is also supported by a study showing attenuation of inflammation in MRL/lpr mouse mesangial cells via inhibition of the PI3K/Akt pathway by EGCG [41]. Taken together, the observed dampening of the signaling pathways following EGCG administration is likely reflecting the reduced inflammation and oxidative stress. Whether these signaling changes also lead to altered cell survival warrants further experimental evaluation.

The EGCG dosage used in our study is relatively low (50 and 25 mg/kg BW/day) compared with other studies where the daily dosage varied from 50–120 mg/kg BW [15, 16, 20]. If extrapolated to human, a 70 Kg person would ingest 1750 mg of EGCG/day (based on the dosage of 25 mg/kg BW/day). According to 2007 USDA report on flavonioid contents (including EGCG) of different food products, one cup of brewed green tea contains, on average, 180 mg of EGCG (http://www.ars.usda.gov/nutrientdata). Thus, 10 cups of tea per day would provide sufficient EGCG dosage for a 70 Kg person.

In summary, our data suggest that EGCG ameliorates biochemical and histologic abnormalities in mice with crescentic GN. The salutary effects of EGCG are likely mediated by its anti-inflammatory and anti-oxidative properties with multiple targets including activating Nrf2-ARE and PPARr pathways and damping AKT/ERK/NF-kB pathways to reduce oxidative stress and inflammation as well as activating SIRT1 to facilitate tissue repair and regeneration. Clinical trials are needed to determine the potential utility of EGCG as a therapeutic agent for the treatment of immune-mediated glomerulonephritides and other immune-mediated diseases in humans.
